# Using freeze-preventive cold boxes in rural Nepal: A study of equipment performance, acceptability, system fit, and cost

**DOI:** 10.1016/j.jvacx.2024.100467

**Published:** 2024-02-28

**Authors:** Sandeep Kumar, Pat Lennon, Surendra Uranw, Tessa Fielding, Mercy Mvundura, Adam Drolet, Steven Diesburg, Arindam Ray, Sagar Dahal, Bibek Lal, Joe Little, Satyabrata Routray

**Affiliations:** a PATH, New Delhi, India; b PATH, Seattle, WA, USA; c Bill & Melinda Gates Foundation, India Country Office, New Delhi, India; d B.P. Koirala Institute of Health Sciences, Dharan, Nepal; e Government of Nepal, Family Health Division, Ministry of Health and Population, Kathmandu, Nepal

**Keywords:** Freeze-preventive cold box, Vaccine cold chain, Cold chain equipment, Immunization, Vaccine freezing, Vaccine delivery

## Abstract

We conducted a field evaluation using qualitative and quantitative methods to assess freeze prevention of vaccines transported and stored in a recently developed, World Health Organization–prequalified freeze-preventive cold box (FPCB) as compared to currently used standard cold boxes (SCBs). The study assessed the FPCB’s practical use, health worker acceptance, health system fit (including cost considerations), and challenges faced by health workers in variable conditions and geographical settings. The evaluation took place in five health facilities across hilly and plains districts of Nepal in two phases: Phase 1 involved FPCBs in simulated use alongside SCBs. In Phase 2, actual vaccines were used in the FPCBs. The study gathered quantitative data from logbooks and electronic temperature monitors placed inside and outside the cold boxes. Qualitative data were collected from health workers, cold chain personnel, and immunization program managers involved in the vaccine cold chain at multiple levels. No damage, durability issues, or freezing incidents were observed when using FPCBs, but two incidents of freezing occurred when using SCBs. FPCBs also took longer to cool down than SCBs. Participants mostly found the FPCB to be safe and user friendly for vaccine transportation and short-term storage. Advantages of the FPCB as compared to the SCB include its ability to minimize vaccine wastage, to keep freeze-sensitive vaccines safe (the average value of freeze-sensitive vaccines transported per shipment was $1,704), and to ease preparation through elimination of the need to condition ice packs. Procurement price ranges for FPCBs overlap those for SCBs. Disadvantages of the FPCB include its greater size and weight, which require more personnel and vehicles during transportation. This suggests that lighter and smaller FPCBs would be more effective and acceptable for the Nepal immunization program and other, similar immunization programs conducted globally.

## Highlights

• Freeze-preventive cold boxes successfully prevent vaccine freezing during transport.
• They simplify cold box preparation and use, as the ice packs are not conditioned.
• They prevent water from accumulating around vaccine vials and secondary packaging.
• They are larger and heavier than standard cold boxes and harder to transport.

## 1 Introduction

Vaccines lose potency over time, and this loss is temperature dependent. The World Health Organization’s (WHO’s) guidelines for vaccine handling recommend specific storage conditions to ensure quality is maintained throughout the vaccine life cycle—from production through storage, transportation, and use [1]. Each step is a critical link in the vaccine cold chain, where considerable effort is put into ensuring vaccines are stored within the recommended temperature range of above 0 °C to below + 10 *°*C.

Vaccine vial monitors have been available for decades to show when vaccines are exposed to heat, yet there remains no vial-level indicator for freezing. The presence of aluminium salt adjuvants in liquid vaccines increases the immunogenicity and efficacy of the vaccine antigen but also increases the vaccines’ freeze sensitivity: freezing can irreversibly damage these vaccines, reducing potency and compromising protective immunogenicity in recipients [2], [3], [4], [5]. This is a concern for many vaccines used in the Expanded Program on Immunization, such as diphtheria-tetanus-pertussis, hepatitis B, *Haemophilus influenzae* type b, pneumococcal conjugate, and inactivated polio vaccines. The importance of protecting vaccines from freezing will become even more pressing globally as introductions of new vaccines put pressure on already weak vaccine supply chain systems [6].

In Nepal, cold boxes are primarily used to transport vaccines from higher cold chain points to health posts. The time required for freeze-sensitive vaccines to freeze depends on the number of doses in the vial (the greater the volume, the longer the time) and the temperatures to which they are exposed. Although several studies conducted in low-, middle-, and high-income countries have reported exposure of vaccines to freezing temperatures at all levels of the health system, the majority of these exposures occurred during transport in cold boxes (portable boxes used mainly in vehicles) and vaccine carriers (smaller units carried by health workers) to lower levels of the health system [5], [7].

Vaccine freezing can lead to either closed-vial vaccine wastage, which has financial implications for immunization programs as it increases vaccine procurement costs, or in cases of undetected freezing, likely leads to a loss of vaccine potency and efficacy. Closed-vial wastage can also waste lifesaving vaccines and increase the risk of stockouts, which can negatively affect timely vaccination and coverage. Freeze prevention at the equipment level mitigates these risks and eliminates the need for shake testing or vial-level freeze indicators, which have not yet proven cost-effective nor been implemented comprehensively.

To keep vaccines cold, both standard cold boxes (SCBs) and vaccine carriers rely on conditioned (i.e., partially thawed) ice packs [8]. If the ice packs are not properly conditioned, they pose a risk to temperature-sensitive vaccines. This step in the cold chain is highly variable because observations indicate that the WHO-recommended practice of conditioning ice packs before use is not routinely followed [7]. Previous studies have consistently documented freezing temperatures in vaccine supply chains [5], [9].

While previous research has explored freeze-preventive vaccine carriers [10], this study represents the first real-world evaluation of a WHO Performance, Quality and Safety (PQS)–prequalified freeze-preventive cold box (FPCB) designed to help address the challenge of vaccine freezing during transport. Cold boxes have a larger capacity than vaccine carriers and are used to transport vaccines between different levels of the supply chain, whereas vaccine carriers are used for outreach and last-mile delivery to health care facilities. The larger capacity means that more vaccines are transported; and therefore, a greater financial loss to the immunization program can result if the vaccines freeze. In 2016, the WHO PQS team developed performance specifications for freeze prevention in cold boxes and vaccine carriers [11]: the temperature in the vaccine storage compartment must remain above 0 °C and below + 10 *°*C with an accuracy of ± 0.5 °C in ambient temperatures of + 15 °C to + 43 °C for a minimum of 48 h for a short-range cold box and 96 h for a long-range cold box [8]. As the result of research by the nonprofit PATH and its manufacturing partners, freeze-prevention technology for passively cooled devices has progressed to the point where it can meet the WHO specifications in laboratory settings. This technology is now publicly available, and in 2020, Qingdao Leff International Trading Company (Leff Trade) became the first manufacturer to receive WHO PQS prequalification for an FPCB. This is the first WHO PQS–certified FPCB to utilize innovative freeze-prevention technology consisting of a specially designed barrier liner between the ice packs and vaccine storage area to prevent direct contact of vaccine vials with ice packs, which is known to result in freezing of the vials. In 2021, PATH, in collaboration with B.P. Koirala Institute of Health Sciences (BPKIHS) in Nepal, conducted a field evaluation of the FPCB. Nepal was selected based on the interest and commitment of the Family Health Division within the Ministry of Health and Population (MOHP) in preventing freezing during vaccine transport, as well as the country’s varied terrain and environmental conditions. The overall objectives were to evaluate whether it performed according to WHO PQS specifications during actual use, was acceptable to end users, and fit well within the Nepalese health system.

The Himalayan terrain poses major geographical challenges for vaccine distribution, transportation, and storage while catering to hard-to-reach health facilities and communities. To address these challenges and limitations within the country’s health infrastructure, the study outcomes will inform or strengthen the cold chain/vaccine management system within its overall health system. In 2015, Nepal shifted from a highly centralized system to a more decentralized model under federalism and has faced both structural and operational issues, including infrastructural weakness, shortage of skilled staff, health worker absenteeism, poor accountability, delays in procurement, and lack of coordination [12]. To improve immunization coverage, Nepal has dedicated significant resources to enhance the service delivery infrastructure of its national immunization program [13], and vaccination coverage varies considerably across its diverse and geographically dispersed population [14], [15]. By effectively reaching underserved and widely dispersed populations, interventions can significantly boost vaccine coverage in Nepal and globally [13], [14]. Even in urban areas, factors like transport costs and access can lead to vaccination dropouts [14].

In Nepal, vaccines are typically collected and distributed from regional and district cold chain points to health posts once per month for routine immunizations. For vaccination campaigns, additional trips may be required. Generally, two to three cold boxes are used per trip. The vaccines are transported to health facilities by either the alternate vaccine delivery (AVD) system or by health workers. AVD personnel are often local persons engaged in this activity on a part-time basis. These workers use cold boxes or vaccine carriers in their own vehicles to collect and transport vaccines, whereas health workers use hospital-owned vehicles to do the same. The monthly vaccine supply is stored in refrigerators at the health facilities. From these facilities, the vaccines are distributed to health posts in cold boxes or vaccine carriers based on need. Health workers subsequently take the vaccines in vaccine carriers directly to communities during vaccination outreach sessions.

## 2 Materials and methods

### 2.1 Characteristics of the device

**Fig. 1:**
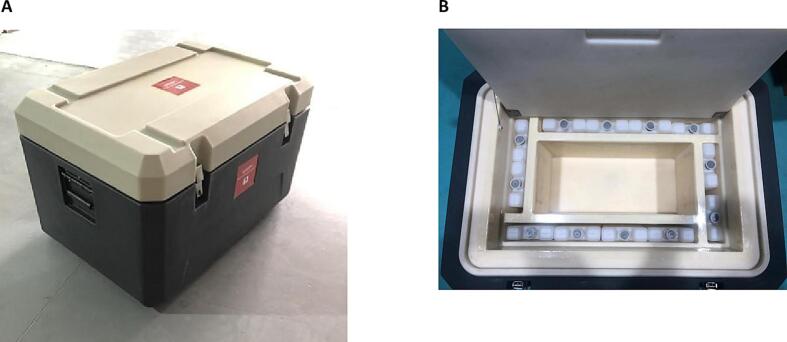
Leff Trade freeze-preventive cold box model FFCB-15L: (A) outside view and (B) inside view.

This study evaluated the Leff Trade FPCB (model FFCB-15L, WHO PQS code E004/057, China) [16] (Fig. 1), which uses chlorofluorocarbon-free (CFC-free) polyurethane as the insulating liner and high-density polyethylene (HDPE) as the external material. Polyurethane foams are widely used, highly insulative materials that are now commonly made using CFC-free, low global warming–potential foaming agents that comply with the Montreal Protocol on Substances that Deplete the Ozone Layer. HDPE is commonly used in large, polymer products like cold boxes, and is robust and fit for purpose. The liner serves to buffer and absorb the cold from 21 frozen ice packs (model WP-0.6L, China) while simultaneously maintaining the vaccine storage compartment within the recommended temperature range of above 0 °C to below + 10 *°*C. Within the liner between the ice packs and the vaccine storage compartment, which includes an internal opening, top, and walls, there is a combination of a liquid material and the insulating foam. The foam both insulates, providing a temperature gradient, and helps to slow down how quickly heat is transferred between the ice packs and the vaccine storage compartment to allow the liquid enough time to release heat through its phase change and effectively condition the ice packs in the cold box to safe temperatures.

The vaccine storage dimensions in the FPCB are 41.5 cm X 18.5 cm X 20.0 cm. The storage volume is 15.4 L. The empty weight is 24 kg; the weight fully loaded with vaccines and ice packs is 49.9 kg. The FPCB is available for procurement through the UNICEF Supply Division. The storage volume of the SCB used in this study is 15 L. The empty weight is 12.2 kg; the weight fully loaded with vaccines and ice packs is 33.9 kg.

### 2.2 Site selection

PATH selected Nepal as the study site based on the country’s range of environmental conditions and terrains and the interest and commitment of the MOHP Family Health Division in preventing vaccine freezing during transport. In consultation with national, regional, and district health authorities, PATH chose two rural districts (plains and hilly), both served by one regional cold chain point, and within each district, chose one district-level cold chain point. In addition, two primary health centers (PHCs) were selected in the plains district and one in the hilly district. Inclusion of these districts, each exemplifying a unique terrain type, was important for understanding the full range of usability and functionality of the FPCB. Each facility received one FPCB; thus, a total of five FPCBs were transported to Nepal. At the end of the pilot, the FPCBs were donated to the MOHP.

### 2.3 Study objectives and design

**Fig. 2:**
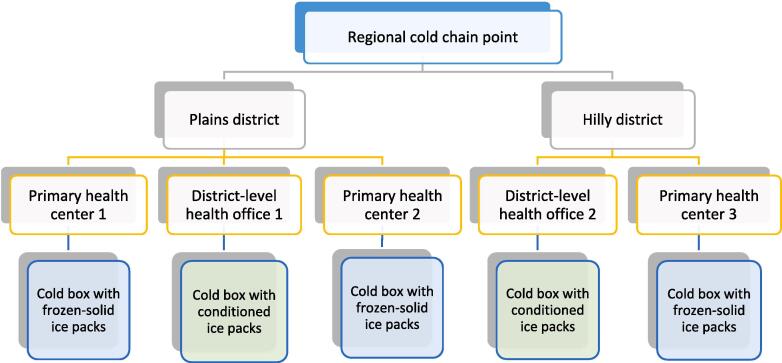
Study locations and ice pack conditions.

The study objectives were to determine whether the WHO PQS–prequalified FPCB, which had performed well in the laboratory, would, in a real-world setting, (1) perform according to the WHO PQS specifications for FPCBs; (2) be acceptable to end users when compared to SCBs; and (3) fit well within the health system, including cost considerations. The study had two phases: Phase 1 (simulated use) and Phase 2 (actual use). During both phases, the two district-level facilities were instructed to condition the ice packs, and the three PHCs were instructed not to condition the ice packs (Fig. 2).

Working in collaboration, PATH and BPKIHS used quantitative and qualitative approaches to conduct the field evaluation. One block monitor per district was hired to support the regional and district cold chain handlers (CCHs); the block monitors collected the study’s quantitative data, supervised the day-to-day activities, and analyzed and sent the collated data for analysis by a data analyst. (One block is equivalent to a health facility administrative area.) The block monitors reported to the principal investigator at BPKIHS, who reported to the PATH study team. The evaluation was conducted with the typical vaccine delivery mechanisms, either AVD personnel or health care staff, to understand whether the larger, heavier FPCBs would have an impact on acceptability and system fit. A qualitative researcher at PATH conducted the qualitative interviews, and another did the data analysis.

### 2.4 Phase 1 (simulated use)

In Phase 1, two types of cold boxes were used simultaneously: the SCB and the WHO PQS–prequalified Leff Trade FPCB. Each FPCB was externally labeled as “For Pilot Purpose Only and Not for Human Use”. Inside were simulated vaccines—real vaccines externally marked with a cross mark (×) and labeled as “Not for Human Use”. In the event of a vaccine shortage during the study period, real vaccines were not used as simulated vaccines, but rather glass vials were filled with appropriately cooled water. As per study procedures, participants kept an inventory of the number of glass vials provided per facility and any breakage of vials. At the completion of Phase 1, the project team (BPKIHS and PATH) discarded all vials as per MOHP waste disposal guidelines.

The CCH for each block prepared the FPCBs with ice packs as per the study protocol: either frozen solid (at or below –20 °C) or conditioned. Instructions for conditioning ice packs were to leave the packs outside the freezer for 30 to 60 min (depending on room temperature) or until the ice was slushy when shaken. In Phase 1, the CCHs ensured that each health facility used their SCB for actual vaccine transport and distribution, with minimal modifications or process changes for the study (i.e., inclusion of one temperature monitoring device). Thus, the mass of the vaccine load transported in these SCBs was not controlled or recorded as part of this study. Facilities were instructed to report the size and quantity of ice packs used in the SCBs.

FPCBs were transported along with an SCB during transport of vaccines from the regional/district level to the health facilities. The facilities were instructed to use a test load in the FPCBs consisting of 0.06 kg of water per liter of vaccine storage capacity, which is the ratio used for laboratory verification testing in the WHO FPCB protocol corresponding to a thermally minimal vaccine load. This equates to a test load of approximately 1 L of water (0.924 L). Facilities were instructed to use a complete set of 21, 0.6 L ice packs in all FPCBs during transport. BPKIHS and PATH verified the simulated vaccine vials reached the identified health facilities and were stored as per MOHP guidelines. PATH supported the block-level officials in developing an AVD plan to account for the delivery of additional cold boxes to each identified health facility/PHC.

Once at the facility, auxiliary health workers and auxiliary nurse midwives placed the temperature monitoring devices (described in a forthcoming section) inside the SCBs and in two locations inside (top and bottom) and one location outside the FPCBs (attached to the side handle). To mimic usage of the SCB during storage and handling of vaccines, the FPCB was opened at the health facility each time the SCB was opened. During vaccination sessions, the health workers were required to open the FPCB a defined number of times and duration at each site. Block monitors downloaded the internal temperature readings of the SCBs and FPCBs (top and bottom) as well as the external, ambient temperature readings of the FPCBs. This pilot test was done for approximately one month of field implementation and one month of data analysis.

### 2.5 Phase 2 (actual use)

In Phase 2, the FPCBs were used during storage and transport of vaccines for actual use. In addition, due to inconclusive SCB data from Phase 1, SCBs were included in the first month of Phase 2 data collection, except at district-level health facility 2, where SCBs were used throughout this phase. The extension of SCB use into Phase 2 allowed for sufficient data to be collected and compared with the data from the FPCBs. The CCH for each block, assisted by the block monitor, as needed, prepared the cold boxes with either frozen-solid or conditioned ice packs (21, 0.6 L ice packs in each cold box), as per the study protocol. Again, PATH supported the block-level officials in developing an AVD plan for delivery of the FPCBs to the identified health facilities/PHCs. Electronic temperature monitoring was done, as in Phase 1, to collect the internal FPCB and SCB temperatures and ambient temperatures (from a monitoring device attached to the side handle of each FPCB). Block monitors downloaded all temperature readings once per month. Phase 2 took place over 2 months of field implementation and one month of data analysis. PATH and BPKIHS maintained close coordination with the MOHP Family Health Division to minimize any disruption in the delivery of vaccination services and ensure proper distribution of required vaccines to cold chain points.

In both phases, the study implemented a data quality assurance plan to ensure the validity and accuracy of the data collected. Activities included real-time data validation and supervisor accompaniments, pictures and interview recordings, and verification that all activities were conducted at the right locations.

### 2.6 Ethics and approvals

PATH obtained ethics approval from the Western Institutional Review Board Copernicus Group, BPKIHS Institutional Review Committee, and Nepal Research Council for the overall study. The MOHP Family Health Division granted approval to conduct the field evaluation. Phase 1 results were shared with the MOHP to obtain approval to progress to Phase 2.

### 2.7 Study participants and training

Study participants included regional and district immunization program heads, health workers responsible for managing the cold chain, and others working in the immunization program at the study sites, including AVD personnel. Participants were selected purposively, targeting staff with roles in vaccine storage and transportation. Expanded Program on Immunization records were used for additional information related to vaccine transportation.

Before initiating each phase, all staff involved in routine vaccination at the five health facilities were oriented to the objectives of the study and trained according to the protocol. This training included CCHs, auxiliary health workers, and auxiliary nurse midwives. Government immunization staff—including district immunization officers, block and district medical officers, and logisticians in the two districts—were also oriented. The orientations and trainings were conducted in the Nepali or Hindi language.

All participants involved in project implementation were eligible to provide qualitative input, though participation in the interviews was voluntary. Written consent was obtained in a private setting before the interviews. For the analysis, data were aggregated to remove any risk of linking to specific facilities. Facility names have not been, and will not be, reported in any publication.

## 3 Quantitative data

### 3.1 Quantitative data collection

Objective 1 sought to determine whether the FPCB performed according to WHO PQS specifications; specifically, this objective aimed to measure the ability of the FPCB to maintain a vaccine compartment temperature between 0 °C (±0.5 °C) and + 10 °C (primary indicator) and whether the FPCB malfunctioned in any way (secondary indicator). The study used LogTag® devices to record temperature data and Parsyl Trek devices to record temperature, humidity, and light data. CCHs kept a daily log to record any incidence of damage or the cold boxes not working properly, including cracks, leaks, improper fit/seal of lids, and damage (e.g., if the cold box was dropped or otherwise impacted). Logbook data (hard copies) were regularly shared with the block monitor (monthly, or in the case of any major issues, immediately) and compiled for analysis. See Table 1 for a summary of the data collection methods.

**Table 1:** Quantitative data collection framework.

Method	LogTag model TRIX-8, WHO PQS code E006/006 (New Zealand)	Parsyl Trek model 1.1 (USA)	Manual, written recordings
Description	WHO PQS–prequalified wide-range, multi-trip datalogger that passively records temperatures from –40 °C to + 85 °C.	Smart sensor that records temperature, humidity, and light data.	Logbook with observations of equipment damage or malfunction.
Placement	Inside vaccine storage compartment (Internal 1 at top and Internal 2 at bottom of compartment) and ambient LogTag attached to outside handle of freeze-preventive cold box.	Inside freeze-preventive cold box.	At health facility.
Indicators monitored	Primary indicators: Internal and ambient temperatures.	Primary indicators: Humidity and light. Secondary indicator: Internal temperature.	Secondary indicator: Equipment breakage or malfunction.
Responsible staff	Cold chain handler	Block monitor	Cold chain handler
Method and frequency of recordings	Automatically records temperature every 10 min.	Automatically records temperature, humidity, and light every 10 min.	Daily or as needed.
Method of collection	Block monitors downloaded data from the device once/month after the monthly transportation cycle. Only data for transportation days were counted.	Block monitors downloaded data from the device once/month after the monthly transportation cycle via Bluetooth and sent to the manufacturer’s server. Only data for transportation days were counted.	Shared with block monitor monthly or as needed.
Destination of data	BPKIHS and PATH	Parsyl and PATH	BPKIHS and PATH
Data reviewer	BPKIHS data manager and PATH	Parsyl and PATH	BPKIHS and PATH

BPKIHS, B.P. Koirala Institute of Health Sciences; PQS, Performance, Quality and Safety; WHO, World Health Organization.

Data for other indicators, including human resources, the AVD plan, the vaccine delivery schedule/plan, and vaccine distribution plans, were collected once at the start of field implementation.

### 3.2 Quantitative data analysis

The study recorded and analyzed the following device indicators: (1) internal and ambient temperature readings per cold chain point; (2) mean internal and ambient temperature readings, including standard deviation and 95 % confidence interval; and (3) number and duration of low (below 0 °C) and high (above + 10 °C) temperature excursions. The data manager at BPKIHS consulted with the field block monitors regularly to communicate any issues or considerations related to the quality of the data. At the end of field implementation, the block monitor and data manager conducted a final quality check and compiled the data into a dataset using sorting features to determine whether any information was missing or identify redundant entries. All temperature data were stored in a common repository. The quantitative data were analyzed in Microsoft Excel® for Microsoft 365 (Version 2401, 64-bit).

### 3.3 Qualitative data

#### 3.3.1 Qualitative data collection

Objective 2 was to determine whether FPCBs were acceptable to end users as compared to SCBs. Following each project phase, feedback on acceptability of the FPCB was collected via in-depth interviews using a paper-based, semi-structured questionnaire that included both five-point Likert scale and open-ended questions. Responses were handwritten on the paper-based questionnaires, and all interviews were audio recorded. AVD personnel and health workers were asked about their experience of learning to use the FPCB as well as its performance/robustness, usability, storage capacity and cleaning, benefits and challenges, and their willingness to use it. District immunization chiefs/officers provided feedback on the health practices, vaccine delivery mechanisms, and challenges in each district. Objective 3 was to assess the feasibility of integrating the FPCB into the health system, including cost considerations. Interviews were conducted at the end of implementation for each phase. In addition, two government district immunization officers were interviewed in each district to understand the challenges of vaccine distribution and introduction of new cold chain technology into the health system (Table 2). All study facilities were selected as participating sites for the qualitative interviews. The participant sample size was based on qualitative methods, with the goal of being descriptive and purposive rather than hypothesis driven.

**Table 2:** Qualitative feedback framework.

Personnel	Sample size	Description	Focus of post-questionnaire/interview
District immunization chief/officer	2	Responsible for overall district immunization program and regular analyses of Expanded Program on Immunization performance; provides technical guidance to concerned staff on vaccine and cold chain management.	Current health practices and challenges in their district.
Cold chain handler at facility level	5	Manages cold chain at the facility level; ensures adequate vaccine and logistics availability for vaccination session sites; ensures effective distribution of vaccines and use of alternate vaccine delivery system.	Freeze-preventive cold box as compared to existing standard cold box: Overall acceptabilityDurability/user friendliness (lid/fit and closure/hinges) Ease of cleaning, cleaning practices, and the time required to cleanEase of vaccine access (placing in and removing vaccines from cold box) Time taken to prepare the cold box before transportation and distribution Weight/size Handling/transportation Vaccine storage capacityLearnability/ease of training
Alternate vaccine delivery personnel/ assistant	5	Supports cold chain handlers at either regional, district, or primary health center level in transporting cold boxes to lower-level health facilities; responsible for cleaning, handling, and transporting the equipment.	Weight, size, and durability of the cold box and overall ease of handling the cold box during transportation and storage.
Total sample size	12		

The study also estimated the value of freeze-sensitive vaccines protected from exposure to freezing temperatures through the use of FPCBs. Data on the number of vials per antigen transported to health facilities in cold boxes during each transportation cycle were used for this analysis; each vaccine transported was classified as freeze sensitive or not, and its value was estimated based on UNICEF vaccine price data [17]. Costing data were collected at the end of the study.

#### 3.3.2 Qualitative data analysis

Written feedback and audio recordings were transcribed and translated to English. Data were entered and cleaned in Microsoft Excel data tables. A thematic approach was used to analyze qualitative acceptability data from the open-ended questions. Responses were manually assessed across acceptability factors (ease of learning, performance, robustness, usability, capacity and cleaning, benefits and challenges, and suggestions) and respondent type to generate themes and patterns from the analysis (see Table 12). Likert responses were aggregated by question and respondent type to generate frequencies and proportions, and used in combination with qualitative themes to generate a more comprehensive understanding of the findings. All data were stored in password-protected computers with access given to the principal investigator and project data analysts/program staff. Feedback from district immunization chiefs/officers was used internally by the project team to ensure program alignment with district guidelines and health practices and to guide the project implementation. Responses from CCHs/assistants are presented by phase below.

## 4 Results

### 4.1 Phase 1 quantitative data

During Phase 1, a large portion of the internal temperature readings from the top internal FPCB LogTags had to be discarded due to incorrect placement: the LogTags were touching the ice packs (inside the FPCB but outside the vaccine storage area), thereby providing incorrect readings. The temperature profile of these readings and speed of temperature change indicated direct contact with an ice pack, rather than with the physically separated, internal vaccine storage compartment. In use, vaccines cannot be physically stored in this location, in direct contact with ice packs. LogTag data were not considered valid if they demonstrated general trends that unmistakably did not match the placement location in comparison to laboratory field testing data of the same cold boxes, or if logbooks indicated incorrect placement or error. This occurred at the district-level health offices; hence, only data from the three PHCs were considered during Phase 1. Furthermore, the three locations reporting usable data had all been instructed to use frozen-solid ice packs; thus, no data on conditioned ice packs are available for Phase 1. Taking this into account, a total of 44 temperature readings from the top internal LogTags and 98 temperature readings from the bottom internal LogTags were noted during Phase 1 for the FPCBs, along with 55 temperature readings from the LogTags in the SCBs. Logbook data confirm that PHC1 loaded the SCB with 13, 0.6 L ice packs in February and 11, 0.6 L ice packs in March. PHC2 loaded the SCB with 10, 0.6 L ice packs in both the February and March outreach sessions. PHC3 loaded the SCB with 16, 0.4 L ice packs in February and 14, 0.4 L ice packs in March. In terms of low temperature excursions, two readings from the SCBs were below 0 °C, representing 3.6 % of the analyzed SCB data. There were no low temperature excursions in the FPCBs, yet both types of equipment had high temperature excursions: in the FPCBs, 21 (47.7 %) of the top internal LogTag readings and 76 (77.6 %) of the bottom internal LogTag readings were above + 10 °C, and in the SCBs, 22 (40 %) of the readings were above + 10 °C (Table 3).

**Table 3:** Phase 1 internal temperature readings in freeze-preventive and standard cold boxes by temperature range.

Temperature	Freeze-preventive cold box		Standard cold box
	Internal 1 (top LogTag) readings	Internal 2 (bottom LogTag) readings	Internal 1 readings
	No. (%)	No. (%)	No. (%)
<0°C	0 (0)	0 (0)	2 (3.6)
0 °C to + 10 °C	23 (52.3)	22 (22.4)	31 (56.4)
>+10 °C	21 (47.7)	76 (77.6)	22 (40.0)
Total readings	44	98	55

Session lengths in Phase 1 ranged from approximately 2 to 3 h (Table 4). Some sessions were not recorded, and some data were removed from analysis, as noted, due to incorrect placement of the temperature monitors. Some FPCB sessions during Phase 1 simulated usage consisted only of ice pack loading and cooldown, without actual vaccine placement. These sessions still represented the expected temperature profiles and were therefore included in the analysis.

**Table 4:** Phase 1 distribution and duration of internal temperatures for freeze-preventive cold boxes and standard cold boxes.

	Block/Health post		Plains		Hilly
			PHC1	PHC2	PHC3
	Number of sessions		2	2	2
	Average length of sessions (minutes)		150	128	178
	Total length of temperature data analyzed (minutes)		300	256	357
FPCB – Internal 1	Number of readings	<0°C	0	0	0
		0 °C to + 10 °C	4	7	12
		>+10 °C	9	5	7
	Length of high temperature excursions (minutes)		80	40	60
FPCB – Internal 2	Number of readings	<0°C	0	0	0
		0 °C to + 10 °C	0	0	22
		>+10 °C	31	27	18
	Length of high temperature excursions (minutes)		300	260	170
SCB	Number of readings	<0°C	1	1	0
		0 °C to + 10 °C	5	19	7
		>+10 °C	9	0	13
	Length of high temperature excursions (minutes)		80	0	120

FPCB, freeze-preventive cold box; PHC, primary health center; SCB, standard cold box.

**Fig. 3:**
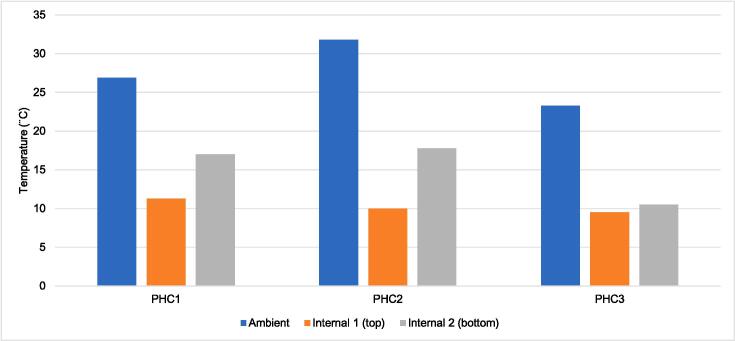
Phase 1 freeze-preventive cold box average temperature readings by LogTag location and health facility. PHC, primary health center.

In Phase 1, higher average ambient temperature readings generally correlated with higher average internal temperature readings in the FPCBs (Fig. 3).

### 4.2 Phase 2 quantitative data

Due to a lack of district logbook data in Phase 2, we were unable to confirm that ice packs were conditioned, or for how long they were conditioned, at the district facilities. Since the protocol specified conditioned ice packs, it was assumed throughout the analysis that this took place. This assumption implies that the worst-case scenario for cooldown rates and best-case scenario for freeze prevention would be represented, in comparison to non-conditioned ice packs. A complete set of 21, 0.6 L ice packs were loaded into each FPCB. Logbook data specified the quantity of various vials transported in the FPCBs during Phase 2, which spanned a range of vaccine vials for each of the 12 vaccine types routinely used during outreach. During the November outreach, PHC1 transported 1,237 vials and PHC2 transported 570 vials. PHC3 transported a total of 172 vials in the December outreach. Vaccines were not scheduled to be delivered or collected in these months at the remaining two PHCs. Not a single incident of freezing was noted in Phase 2 for the FPCBs. However, 19 freezing temperatures (1 % of readings) did occur in the SCBs. In addition, as in Phase 1, both types of equipment experienced high temperature excursions: in the FPCBs, 152 (1.7 %) of the top internal LogTag readings and 372 (4.7 %) of the bottom internal LogTag readings were above + 10 °C, and in the SCBs, 300 (15 %) of the readings were above + 10 °C (Table 5).

**Table 5:** Phase 2 internal temperature readings in freeze-preventive and standard cold boxes by temperature range.

Temperature	Freeze-preventive cold box		Standard cold box
	Internal 1 (top LogTag) readings	Internal 2 (bottom LogTag) readings	Internal 1 readings
	No. (%)	No. (%)	No. (%)
<0°C	0 (0.0)	0 (0.0)	19 (1.0)
0 °C to + 10 °C	8,561 (98.3)	7,461 (95.3)	1,679 (84.0)
>+10 °C	152 (1.7)	372 (4.7)	300 (15.0)
Total readings	8,713	7,833	1,998

Cold box sessions in Phase 2 typically lasted for one day and were used to transport vaccines and ice packs to the facility level. However, cold boxes were occasionally used to store vaccines, resulting in session lengths of greater than one day (Table 6).

**Table 6:** Phase 2 distribution and duration of internal temperatures for freeze-preventive cold boxes and standard cold boxes.

	Block/Health post		Plains			Hilly	
			PHC1	DH1	PHC2	DH2	PHC3
	Number of sessions		3	3	4	6	4
	Average length of sessions (hours)		95.7	128.7	48.6	31.5	148.9
	Total length of temperature data analyzed (hours)		287.0	386.2	194.2	189.0	595.7
FPCB – Internal 1	Number of readings	<0°C	0	0	0	0	0
		0 °C to + 10 °C	1,690	1,299	1,201	1,093	3,278
		>+10 °C	35	15	49	40	13
	Length of high temperature excursions (minutes)		340	140	480	390	120
FPCB – Internal 2	Number of readings	<0°C	0	0	0	0	0
		0 °C to + 10 °C	524	1,398	1,232	1,035	3,272
		>+10 °C	38	191	35	101	7
	Length of high temperature excursions (minutes)		370	1,900	340	1,000	60
SCB internal	Number of readings	<0°C	12	0	N/A	7	N/A
		0 °C to + 10 °C	29	593	N/A	1,057	N/A
		>+10 °C	249	0	N/A	51	N/A
	Length of high temperature excursions (minutes)		2,480	0	N/A	500	N/A

DH, district-level health facility; FPCB, freeze-preventive cold box; PHC, primary health center; SCB, standard cold box.

**Fig. 4:**
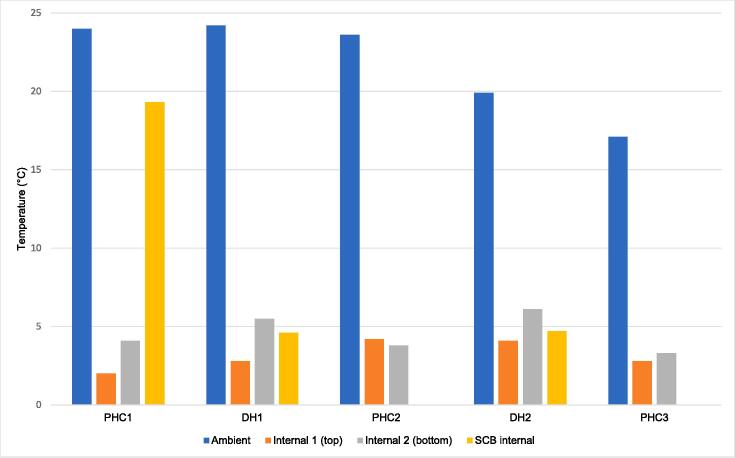
Phase 2 freeze-preventive cold box (Internal 1 and Internal 2) and standard cold box average temperature readings by LogTag location and health facility. DH, district-level health facility; PHC, primary health center; SCB, standard cold box.

For the PHCs in Phase 2 that collected internal temperature readings for SCBs, the average temperatures were similar to the FPCBs, with the exception of PHC1, where average temperatures were significantly higher in the SCBs (Fig. 4). The average internal temperatures of the cold boxes at each facility location represent varying total session lengths. Two facilities, PHC2 and PHC3, did not report any SCB data during Phase 2.

### 4.3 Phase 1 qualitative data

#### 4.3.1 Standard cold box

During the Phase 1 simulated usage of the FPCB along with actual usage of the SCBs, respondents (n = 5) noted a number of advantages of the SCBs, including being lightweight (100 %), having long holdover periods (40 %), easily carried and transported (40 %), and familiar to Nepalese cold chain staff (20 %). Yet all respondents also stated they faced challenges with the SCBs. These included the time required to condition the ice packs (100 %), accumulation of water droplets at the bottom of the cold box (80 %), vaccine vial labels coming off due to this moisture in the cold box (20 %), preparing the cold box (20 %), and concerns about the potency of vaccines if they came into contact with the ice packs (20 %). See Table 7.

**Table 7:** Descriptive feedback on advantages and challenges of standard cold boxes.

	Feature	Response rate (n = 5)
Advantages of SCBs	Lightweight	100 % (5/5)
	Long holdover period	40 % (2/5)
	Easily carried and transported	40 % (2/5)
	Familiarity of device to health staff	20 % (1/5)
Challenges with SCBs	Time required to condition ice packs	100 % (5/5)
	Water droplet accumulation	80 % (4/5)
	Vaccine vial label damaged	20 % (1/5)
	Preparing the cold box	20 % (1/5)
	Vaccine potency concerns due to ice pack contact	20 % (1/5)

SCB, standard cold box.

#### 4.3.2 Freeze-preventive cold box

The FPCB was seen in Phase 1 as either very (60 %) or moderately (40 %) acceptable by respondents (see Table 8). The overall ease of use was noted by all respondents as the top feature.

**Table 8:** Phase 1 acceptability of the freeze-preventive cold box compared to standard cold boxes.

Question	Very acceptable	Moderately acceptable	Neutral	Moderately unacceptable	Very unacceptable	No response
In your opinion, how acceptable, overall, was this FPCB compared to the SCB? (n = 5)	60 % (3/5)	40 % (2/5)	0 %	0 %	0 %	0 %

FPCB, freeze-preventive cold box; SCB, standard cold box.

All respondents stated the new FPCB offered more benefits than the SCB for vaccine transportation and outreach activities. Reasons included conditioning not required, which saved time (100 %); greater vaccine protection and reduced wastage (60 %); separate vaccine and ice pack compartments, which eliminated wet vaccine cartons and vials (40 %); and fit for long-term storage in hilly areas (20 %), where access to and availability of cold chain equipment and infrastructure is more of a challenge.

All respondents believed the capacity of the new FPCB was sufficient for transporting vaccines for all routine vaccination outreach sessions and to a lesser degree for vaccination campaigns, noting that the smaller storage volume of the new FPCB compared to the SCB would mean an increase in the number of FPCBs required to transport similar vaccine stock quantities, especially during campaigns. Across the Phase 1 interviews, all respondents consistently highlighted two principal challenges with the FPCBs: weight and size.

Respondents noted that learning to use the FPCB was either very easy (60 %) or moderately easy (40 %). There was general confidence (60 % “moderately confident” and 40 % “very confident”) that other health workers could learn to use the FPCB with proper training (see Table 9).

**Table 9:** Phase 1 confidence in other health workers’ ability to follow instructions for using the freeze-preventive cold box.

Question	Very confident	Moderately confident	Neutral	Moderately concerned	Very concerned	No response
How confident are you that other health workers will be able to follow the instructions? (n = 5)	40 % (2/5)	60 % (3/5)	0 %	0 %	0 %	0 %

All respondents (100 %) mentioned that no water or droplets accumulated in the vaccine compartment of the FPCB; this was attributed to the separation of the vaccine and ice pack compartments. No component of the FPCB was reported to have broken, become damaged, or malfunctioned during the pilot. One CCH noted difficulty in closing the latch of the FPCB, due to its design.

Overall, 40 % of respondents in Phase 1 would prefer to use the SCB, and 60 % would prefer to use the FPCB.

#### 4.3.3 Phase 2 qualitative data

Overall, in Phase 2, the new FPCB was seen as either very (80 %) or moderately (20 %) acceptable by respondents, and all respondents (100 %; n = 5) viewed the FPCB as providing more benefits than SCBs (see Table 10).

**Table 10:** Phase 2 acceptability of the freeze-preventive cold box compared to standard cold boxes.

Question	Very acceptable	Moderately acceptable	Neutral	Moderately unacceptable	Very unacceptable	No response
In your opinion, how acceptable, overall, was this FPCB compared to the SCB? (n = 5)	80 % (4/5)	20 % (1/5)	0 %	0 %	0 %	0 %

FPCB, freeze-preventive cold box; SCB, standard cold box.

The ease of use, and specifically eliminating the need to condition ice packs, was voiced by all respondents as an advantage of the new FPCB compared to other cold boxes. All respondents noted the FPCB had sufficient storage capacity to hold the vaccines needed for typical routine vaccination sessions and/or vaccination campaign outreach events, although district vaccine stores may require three to four cold boxes for storage purposes. (See Table 11.).

**Table 11:** Descriptive feedback on advantages and challenges of the freeze-preventive cold box.

	Feature	Response rate (n = 5)
Advantages of FPCB	No ice pack conditioning required	100 % (5/5)
Long holdover period	Reduces risk of vaccine freezing/wastage	80 % (4/5)
	Time savings	40 % (2/5)
	Vaccine cartons do not become soggy or wet	40 % (2/5)
Challenges with FPCB	Size and weight of FPCB	100 % (5/5)

FPCB, freeze-preventive cold box.

**Table 12:** Phase 2 qualitative data results.

Facility	PHC1	DH1	PHC2	DH2	PHC3
Introduction					
Advantages of SCB	Weight, easy to carry (even by one person), easy to clean, easy to transport.	Easy to use and lightweight.	Weight, thus no additional person is required during transportation.	Long holdover time, light weight.	Lightweight and easy to carry/transport.
Do you face challenges with the SCB?	Yes	Yes	Yes	Yes	Yes
If so, what challenges?	Water droplet accumulation due to ice packs being in direct contact with the vaccine cartons and affecting the boxes (becoming soggy); time spent conditioning ice packs.	Time spent conditioning ice packs; vaccine boxes becoming soggy and labels coming off due to water.	Time spent conditioning ice packs; water accumulation at bottom of cold box.	Water droplet accumulation, as it affects vaccine boxes/labels.	Water droplets and time spent conditioning ice packs.
Ease of learning					
Were you provided training on use of the FPCB?	Yes	Yes	Yes	Yes	Yes
How easy or difficult was it to learn to use the FPCB?	1—Very easy	1—Very easy	1—Very easy	1—Very easy	1—Very easy
How confident are you that other health workers will be able to follow the instructions?	1—Very confident	1—Very confident	1—Very confident	1—Very confident	2—Moderately confident
Do you need any additional training on use of the freeze-preventive cold box?	No	No	No	No	No
What topics should be covered in the training?	Trainings might be required for new health workers; all health workers should be trained on the new technology/cold box usage and maintenance.	The FPCB is easy to use and even an untrained person can use or prepare it, as conditioning is not required.	Cleaning at the bottom of the ice pack placement section is difficult, hence more focus during training on cleaning practices.	Trainings to be provided to additional health staff posted at the cold chain point or health facility.	Easy to use but training will be required for new health workers.
Performance					
In your opinion, how did the FPCB perform in maintaining temperatures and preventing freezing?	No incident of vaccine wastage due to heat exposure (e.g., VVM change/unusable stage) or freezing incident happened. Freezing mostly happens when frozen ice packs come in contact with the vaccine vials directly, and in this cold box, it is not possible, as there is barrier between the vaccine compartment and ice packs.	Validation done by thermometers, LogTags, and other remote temperature monitoring devices helped in validation of temperature performance. Due to short distance to regional store, we regularly check the VVM status and freezing; we check the vials for any form of sedimentation.	By touching inside the FPCB, one can feel the coldness and can comment about the inner temperature. For heat exposure, health workers rely on VVM readings. For freezing, due to barrier between the ice packs and vaccine compartment, vaccines will not go to freezing stage.	Temperature maintenance, based on the condition of ice packs placed in the cold box and also touching inside. LogTags or Berlinger Freeze Tags are also used during transportation. VVMs help health workers in realizing/checking whether vaccines have been exposed to heat. Freeze sensitive is more difficult to assess, as health workers are not usually trained much to do the shake test.	By touching inside and looking at the condition/status of the ice pack: whether it is stony hard or watery. VVM also is an important indicator for heat exposure.
What was the condition like in the vaccine compartment of the FPCB?	No water droplet accumulation noticed in vaccine compartment. Water accumulation/ droplets were noticed in the ice pack compartment, but as the compartments are different and not connected, the water didn’t affect the vaccine compartment. Vaccine vials are usually placed in zipper polybags.	No water droplet accumulation noted, as both ice packs and vaccine compartments are different. Water accumulation noted in ice pack section if ice packs are kept in cold boxes for few days.	No water accumulation or droplets noticed in the vaccine compartment. Still, zipper polybags are being used for vaccine storage.	No water droplet accumulation noted, as both ice packs and vaccine compartments are different.	No water droplet accumulation noted, as both ice packs and vaccine compartments are different.
What was the condition like in the vaccine compartment of the SCB during earlier usage period?	Yes, water accumulation has been noticed multiple times in the SCB and vaccine vials/boxes become soggy and wet, affecting vial labels. It is more in the SCBs as compared to FPCBs.	Yes, water droplets accumulate in SCBs.	Yes, water accumulation has been noticed multiple times in the SCB. It is more noted in SCBs as compared to FPCBs.	Yes, water droplets accumulate in SCBs. Zipper polybags are being provided.	Yes, water droplets accumulate in SCB.
Robustness					
Did any component of the FPCB break, get damaged, or malfunction during the pilot testing?	No	No	No	No	No
Due to the “No” answers above, interview follow-on questions and answers are noted here as N/A.	N/A	N/A	N/A	N/A	N/A
Usability					
In your opinion, how acceptable, overall, was this FPCB compared to the SCB?	1—Very acceptable	1—Very acceptable	1—Very acceptable	1—Very acceptable	2—Moderately acceptable
Can you describe your experience using the FPCB over the past few weeks?	Usage has been very easy due to segregation of the ice packs and vaccine compartment and conditioning not required. It saves time.	Weight being an issue; otherwise, no problems noted.	No problems noted.	Weight and size.	For female cold chain handlers and assistants, it is more difficult to carry due to its weight and very difficult to carry in hilly terrains.
What did you like best about the FPCB?	Preparing the cold box is easy and quick, as ice packs can be placed directly in the cold box, which saves time in summer but also in winter as we don’t have to wait for conditioning of ice packs, and there is no risk of freezing.	Easy to use and prepare and store vaccines in FPCB.	Ease of use and time required to prepare, as conditioning need not be done.	Easy to use, helpful for hilly terrains/areas, and will help prevent vaccine freezing. Easy storage and handling.	Easy to prepare cold box.
What did you like least about the freeze-preventive cold box?	Transportation due to heavy weight and two persons required for transportation and lifting/moving from one place to another. Vehicle hiring support also required, leading to additional expenses.	Heavy weight and two persons are required for transportation and lifting/moving from one place to another. Difficult to transport on bicycle or public transport, hence additional vehicle is required.	Heavy weight and at least two persons are required for transportation and lifting/moving from one place to another.	Transportation is a challenge for health posts, as they have to hire additional vehicle for carrying the FPCB.	Transportation is a challenge, especially in hilly and difficult terrains.
Would you rather continue using the FPCB or SCB?	FPCB	FPCB	FPCB	FPCB	SCB
Why?	[FPCB] prevents freezing and saves time.	[FPCB] due to the benefits and separate cold chain storage space.	SCB is preferred due to its weight, but considering the vaccine freezing point, FPCB is preferred.	[FPCB] due to more benefits and fit for hilly areas for storage and time saving, as conditioning is not required.	[SCB] due to weight issue and easier to carry.
Capacity and cleaning					
In your opinion, is the capacity of the FPCB sufficient to transport vaccines for routine immunization sessions and vaccination campaigns in comparison to the SCB?	Yes	Yes	Yes	Yes	Yes
Please describe.	Sufficient capacity, especially for outreach/routine immunization requirement.	Sufficient capacity available, but for district cold chain point, FPCBs were used during the COVID-19 vaccination campaigns and three to four cold boxes are required as one to two will not suffice.	Sufficient capacity, especially for the health facility.	Sufficient capacity for hilly areas.	Sufficient for hilly areas/health facilities.
How often do you clean the FPCB?	Once a month.	Once a month.	Once a month.	Once a month.	Once a month.
How do you clean it?	Clean cloth and soap.	Clean cloth and soap.	Soap, water, and clean cloth.	Clean cloth, soap, water.	Water, soap, clean dry cloth.
Do you need any additional supplies for cleaning?	No	No	No	No	No
How much time does it take to clean the FPCB?	10 min	10–20 min	10–15 min	10–15 min	10–15 min
Does it take more or less time in comparison to the SCB?	More	Same	More	Same	More
Please describe.	It takes more time, as FPCB has compartments and reaching/cleaning the bottom of compartment takes more time.	N/A	Slightly more time due to compartments and difficulty reaching the bottom of the ice pack section in FPCB.	N/A	N/A
Benefits and challenges					
From your perspective, in comparison to the SCB, would the FPCB offer more benefit or more drawbacks in management of vaccines for outreach?	More benefit.	More benefit.	More benefit.	More benefit.	More benefit.
Please describe.	Benefits in terms of time saving, conditioning not required, vaccine wastage being reduced, and vaccine cartons/vials don’t become soggy and no risk of freezing of vaccines at all.	Conditioning not required, easy to use, and reduction in vaccine wastage.	More benefits in terms of time saving, conditioning not required, vaccine cartons/vials don’t become soggy or wet. From vaccine safety perspective, prefer FPCB, but from personnel and transportation perspective (cost and additional expenses), prefer SCB.	Conditioning not required, easy to use, and reduction in vaccine wastage. Fit for hilly areas for long-term storage.	Easy to prepare without conditioning and separate place for vaccine and ice packs. Only drawback is difficult to carry in hilly terrains.
Did you (or the AVD) face any difficulties while collecting vaccines from the vaccine store and delivering it to the cold chain point?	Yes	No	Yes	No	Yes
What caused the difficulty?	Only challenge was due to weight of the cold box.	N/A, as vehicle was used for transportation, no major issue.	Weight being the main challenge.	N/A, as vehicle was used for transportation, no major issue.	Due to its size and weight, difficulty during transportation, as our district has more hilly terrain.
How did you manage the difficulty?	Two persons had to carry/transport the cold box.	N/A. Additional vehicle was hired for transportation.	N/A. Additional vehicle was hired for transportation.	N/A. Additional vehicle was hired for transportation.	N/A. Additional vehicle was hired for transportation.
Do you feel the weight and size of the FPCB would be a major, minor, or no challenge if you were to use it?	Major	Minor	Major	Minor	Major
Please describe.	Size and weight are the main issues. If weight can be reduced, then it will be easier for one person to use the cold box.	Weight is a factor, but if Nepal Ministry of Health procures in regular system, then the health officials have to use it.	Size and weight are the main issues.	Weight is a factor; it is difficult for one person to manage/transport.	Weight is an issue.
Suggestions					
Do you have any other observations or comments on how to improve the FPCB?	Weight reduction and using same material as SCB.	If size and weight can be reduced with increase in storage capacity of vaccines.	Size and weight to be reduced.	Size and weight to be reduced.	Improvement in size and weight.

AVD, alternative vaccine delivery (personnel); DH, district-level health facility; FPCB, freeze-preventive cold box; N/A, not applicable; PHC, primary health center; SCB, standard cold box; VVM, vaccine vial monitor.

As in Phase 1, all respondents in Phase 2 noted size and weight as the biggest drawbacks of the FPCB. The need for two people to lift and move the box, and the additional vehicles to be hired to transport the boxes, created significant challenges, including added costs and expenses, especially for hilly terrains and locations. Bicycles and public transport were not seen as feasible for transporting the FPCBs.

“As the new freeze-preventive cold box is heavy, even during the pilot and actual usage phase, additional vehicle was hired for the transportation. BPKIHS/project supported the vehicle hiring, but this might be difficult once the health system adopts, as funds are limited. Only issue during the actual usage period is the weight issue during handling.” – Respondent at PHC2

Regarding performance, no incident of vaccine wastage due to total heat exposure (i.e., the vaccine vial monitor reaching an unusable stage) or freezing incident was reported for the FPCBs. All respondents noted there was no water accumulation in the vaccine compartment due to the separate vaccine and ice pack compartments. This separation of compartments was repeatedly noted as addressing a perennial cold box limitation: water accumulation in the vaccine compartment, leading to soggy vaccine cartons and damaged vaccine vial labels. No components of the FPCB were reported to have broken, become damaged, or malfunctioned during the pilot.

Overall, 80 % of respondents stated they would rather continue using the FPCB compared to SCBs (Table 12).

#### 4.3.4 Cost estimates

An FPCB costs from $234 to $262 to procure, whereas an SCB costs from $75 to $1,320 (there are currently more SCBs, hence the larger range). During the evaluation period, an average of approximately 4,000 doses was transported in one cold box per trip (Table 13). The average value of all vaccines transported per shipment was $2,739, of which an average value of $1,704 (62 %) was freeze sensitive.

**Table 13:** Doses and values of vaccines taken per shipment in freeze-preventive cold boxes.

	Average	Minimum	Maximum
Doses taken per shipment	4,059	1,184	7,990
Value of all vaccines taken per shipment	$2,739	$676	$5,499
Value of freeze-sensitive vaccines taken per shipment	$1,704	$360	$3,371
Percentage of freeze-sensitive vaccines taken per shipment	62.0 %	53.0 %	65.0 %

## 5 Discussion

**Fig. 5:**
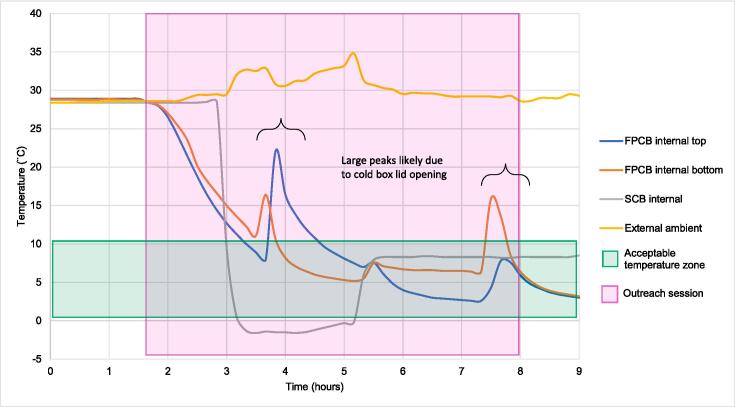
Example of time versus temperature graph illustrating thermal performance of freeze-preventive and standard cold boxes during one session. FPCB, freeze-preventive cold box; SCB, standard cold box.

This study was conducted to validate product performance during actual use. In terms of the primary purpose of the equipment—preventing vaccines from freezing—the FPCB performed to standards. Aside from this, a significant number of high temperature excursions occurred. As can be seen in Fig. 5, the SCBs cooled down much more quickly than the FPCBs. The thermal buffering used to protect the vaccine storage area from freezing temperatures in the FPCBs makes them take longer to cool. For extended outreach times or temporary storage of vaccines off-site or when electric-powered equipment fails, this is not much of an issue. However, during short outreach sessions, this slower cooldown can mean exposure to elevated temperatures for most of the session. Considering the overall degradation of vaccines due to total high temperature exposure, the amount and level of thermal exposure is relatively minimal but can be very noticeable to health workers trained to keep vaccines within the acceptable temperature range of above 0 °C and below + 10 *°*C.

Also notable in Fig. 5 is the relatively long period of freezing in the SCBs, which is not uncommon and can lead to loss of vaccine potency. and therefore provide lower protection than expected if administered to beneficiaries/patients or if detected lead to discarded vaccines. An additional finding was that the upper measurement location in the FPCB was often colder, especially when the equipment appeared to remain closed. It seems this was due to the design of the particular cold box in this study, which has a full sheet of ice packs on the inner lid of the protected vaccine storage area. In standard equipment, it is usually the bottom locations that remain colder than the top, both because the lid opens at the top, with the lid seal being a thermal weak point, and due to natural convection within the storage area.

Despite numerous high temperature excursions, no incident of vaccine wastage due to total heat exposure was reported. There will generally be a short period of high temperatures when vaccines are first placed in cold boxes. As noted, this time period was extended significantly by the FPCBs due to the thermal buffering between the ice packs and the vaccines themselves. Yet the primary advantage of FPCBs over SCBs is prevention of freezing even when fully frozen, non-conditioned ice packs are used. In addition to safeguarding the potency of freeze-sensitive vaccines, the ability to use fully frozen ice packs is expected to simplify logistics, as noted, by reducing the amount of time required by health workers to prepare cold boxes for vaccine distribution. An additional advantage could include a reduced training burden. Further research and development toward smaller and lighter FPCBs relative to their usable storage volume is recommended, as participants continually noted this issue. The tested FPCB model weighs 46 kg when fully loaded per PQS definition, just slightly less than the 50 kg limitation set by WHO PQS. Smaller and more volume-optimized designs could be more fitting for the specific needs and requirements of the Nepalese immunization program. Finally, the cost to procure one FPCB is much less than the value of the vaccines that are prevented from freezing. We saw two freezing events with the SCBs. The occurrence of such events during a limited study duration indicates that investing in FPCBs to prevent freezing of expensive vaccine shipments is potentially a good value for money.

### 5.1 Limitations

Staff were interviewed at the completion of each phase, and responses could have been influenced by recent events (e.g., COVID-19 pandemic). The sample size of 12 participants across five health facilities for the qualitative interviews was not meant to be statistically representative. Furthermore, the study took place across only a few months of the year, and involved only Nepal and its immunization system, so may not represent the diversity of global situations and systems in which these cold boxes might be used. Only a single, new device model was studied as it was the only FPCB prequalified at the time of the study. Responses in this type of study comparing a single, new piece of equipment to older equipment can be biased toward the newness of the equipment with no other equivalently new comparator. Future studies should evaluate the budget impact and cost-benefit of FPCBs to provide evidence on the economic feasibility of implementing this technology within the entire immunization program of a country. These types of analyses could provide insights into the sustainability and long-term benefits of adopting FPCBs within existing immunization programs.

## 6 Conclusion

The development and deployment of freeze-prevention technology for passively cooled vaccine boxes is an important step forward for last-mile distribution of lifesaving vaccines. The Leff Trade FPCB provided valuable freeze protection and minimized preparation time for health care workers by eliminating the need for ice pack conditioning, but also demonstrated significant drawbacks such as its larger weight and size, which made it more difficult to lift and transport. The FPCB also presented a thermal challenge for short-term usage due to its longer cooldown period. Although this heat exposure may not cause actual vaccine wastage, the additional heat exposure is not ideal and may introduce unnecessary concern among health care workers trained to maintain vaccines within the temperature range of above 0 °C to below + 10 °C. Better and newer equipment may address this cooldown issue in the future, but with the FPCB studied here, the more important freeze-prevention characteristic was well addressed. These tradeoffs will need to be carefully considered by country immunization programs. More ergonomic product designs may be better suited to the unique needs of the Nepalese immunization program.

Recommendations for future research and development based on these findings include (1) explicitly modeling, validating in real-world conditions, and publishing estimated vaccine degradation due to any additional heat exposure from demonstrated longer cooldown periods; and (2) further product development that might lead to lighter equipment with more storage space and a shorter cooldown period while still thermally protecting vaccine potency from both freezing and heat exposure.

This work was supported by the Bill & Melinda Gates Foundation, Seattle, Washington, USA (grant number INV-024428). The views expressed herein are solely those of the authors and do not necessarily reflect the views of the foundation.

## CRediT authorship contribution statement

**Sandeep Kumar:** Writing – review & editing, Writing – original draft, Supervision, Project administration, Methodology, Conceptualization. **Pat Lennon:** Writing – review & editing. **Surendra Uranw:** Writing – review & editing, Validation, Supervision, Methodology. **Tessa Fielding:** Writing – review & editing, Formal analysis, Data curation. **Mercy Mvundura:** Writing – review & editing, Formal analysis. **Adam Drolet:** Writing – review & editing, Formal analysis. **Steven Diesburg:** Writing – review & editing. **Arindam Ray:** Writing – review & editing. **Sagar Dahal:** Writing – review & editing. **Bibek Lal:** . **Joe Little:** Writing – review & editing, Resources, Project administration. **Satyabrata Routray:** Writing – review & editing, Methodology.

## Declaration of competing interest

The authors declare that they have no known competing financial interests or personal relationships that could have appeared to influence the work reported in this paper.

## Data Availability

The relevant data are included in the article.
